# Arthroscopic findings of a diagnostic dilemma- hip pathology with normal imaging

**DOI:** 10.1186/s12891-017-1485-5

**Published:** 2017-03-21

**Authors:** Joel Glenn Buikstra, Camdon Fary, Phong Tran

**Affiliations:** 1 Austin Health, Austin Hospital, PO Box 5555, Heidelberg, VIC 3084 Australia; 2 Orthopaedic Surgery, Western Health, Footscray, VIC Australia

**Keywords:** Hip arthroscopy, Magnetic resonance imaging, Image-guided injection, Intra-articular hip pathology

## Abstract

**Background:**

Patients with groin, hip and pelvic pain but normal findings on MRI and minimal changes on x-ray can be a diagnostic problem. This paper looks at the arthroscopic findings of patients who have had hip pain and a positive response to an intra-articular anaesthetic but have non-contributory imaging. We hypothesized that standard MRI’s were missing significant pathology and if there was a response to intra-articular local anaesthesia, pathology found during arthroscopy was likely.

**Methods:**

A retrospective review of all hip arthroscopies performed from March 2011 to January 2015 by two orthopaedic surgeons specializing in hip arthroscopy was conducted to identify patients with clinically suspected intra-articular hip pathology despite a normal MRI report and X-ray. Clinical suspicion of intra-articular hip pathology was confirmed with a positive response to a fluoroscopically guided intra-articular injection of local anaesthetic and corticosteroid. Pathologic findings were collated from the standardised operative notes.

**Results:**

Fifty-three hip arthroscopies performed in 51 patients met the inclusion criteria from a total of 1348 hip arthroscopies performed over a 46-month period. All but one of the 53 (98%) hips had arthroscopically confirmed pathology. Mean patient age was 32.5 years [15 to 67 years] with 40 (78%) females and 11 (22%) males. 92.5% of the hips (49/53) were FADIR (flexion, adduction and internal rotation) positive on clinical examination, giving this test a positive predictive value of 98% (95% CI: 89.31 to 99.67%) for intra-articular pathology.

**Conclusions:**

In patients with a normal MRI without contrast and a positive response (relief of pain) to an intra-articular injection that failed conservative management, there is a 98% chance of intra-articular hip pathology being discovered on hip arthroscopy.

**Electronic supplementary material:**

The online version of this article (doi:10.1186/s12891-017-1485-5) contains supplementary material, which is available to authorized users.

## Background

This study explores the arthroscopic findings in patients with suspected hip pathology and normal hip X-ray (standing anteroposterior (AP) Pelvis, Lateral hip and Dunn views) and Magnetic Resonance Imaging (MRI) without contrast imaging but a positive response to an intra-articular hip injection of a local anaesthetic and corticosteroid. These patients pose diagnostic dilemmas that can be difficult to manage with little literature addressing how to approach the diagnostic workup in this select group. We hypothesized that standard XR and non-contrast MRI miss clinically significant pathology if there was a positive response to intra-articular local anaesthesia.

The diagnostic accuracy of physical examination tests of the hip are unfortunately poor, although combined with a detailed history can often predict the presence of intra-articular pathology [1–3]. The majority of cases require the use of additional investigative and diagnostic tests. Despite the recent advancements in imaging, hip pathology can be missed resulting in progressive symptoms and pathology. Diagnosis may not be made until sufficient damage has occurred that is irreversible (e.g. cartilage loss). Early diagnosis is therefore crucial for early successful management.

Although it has been well established that a negative MRI should not preclude hip arthroscopy if there is high clinical suspicion [4], there is very limited literature looking at this population. MR arthrography remains the more sensitive diagnostic test when compared to MRI for intra-articular hip pathology and in particular labral tears, although there are discrepancies in the literature [5–8].

Local anaesthetic injection for diagnostic purposes with or without corticosteroid for therapeutic purposes is a common practice [9]. According to Jacobson et al. [10] and Kivlan et al. [11], pain secondary to chondral damage and femoroacetabular impingement (FAI) respond particularly well to diagnostic intra-articular local anaesthetic injections. Another study has demonstrated the response to injection is 90% accurate for detecting the presence of intra-articular pathology [1].

## Methods

### Inclusion criteria

We retrospectively reviewed all hip arthroscopies performed from March 2011 to January 2015 by two orthopaedic surgeons specializing in hip arthroscopy. Patients with a normal XR and MRI report, failed conservative management (including activity modification, analgesic medication and physical therapy) and positive response to hip joint preoperative anaesthetic injection were identified from the medical records.

### Imaging

All patients had three routine plain XR views taken; standing anteroposterior (AP) Pelvis, Lateral hip and Dunn views to exclude osteoarthritis, hip dysplasia and FAI as a cause for the clinical suspicion of hip pathology. XR and MRI’s were performed at a range of radiology departments in Melbourne (Australia), and reporting radiologists had a range of experiences although approximately half (25/53 or 47%) had specialist training in musculoskeletal radiology. Of the MRI’s 56% were performed with a 3.0 Tesla (T) scanner, 15% with a 1.5 T scanner and of the remaining 29%, the strength of the magnet was unclear in the clinical database. The surgeons agreed with the reporting radiologist in all cases after reviewing the images themselves during the patient’s following consultation.

### Intra-articular injection

Those patients with unrevealing imaging went on to have a fluoroscopically guided intra-articular injection of corticosteroid and local anaesthetic. Pain relief following the injection was defined by a substantial improvement of pain as indicated by the numerical rating scale (NRS-11), which is a self-reported pain scale from 0 to 10 [12]. Those with pain less than 6 out of 10 prior to injection were excluded. Patients with pain 0 or 1 out of 10 for up to 48 h after the injection were considered to have had a positive result. Additional file 1 demonstrates the pain diary patients were asked to complete at the time of the injection along with verbal instructions.

### Operative findings

Once we identified those that met the inclusion criteria, we looked at the arthroscopic findings by 2 fellowship-trained hip arthroscopists and recorded the details of the pathology found. In particular, we reviewed the operative notes for the presence and details of 3 common intra-articular pathologies- Ligamentum teres (LT) tears, labral tears, and chondral damage. LT anatomy was classified as normal, a partial, complete or degenerative tear [13]. As with Devitt et al. [14], we feel that LT fraying and elongation represent partial tears associated with instability/subluxation and so these two findings were classified as such. Labral tear size and location using a clock-face description [15] were recorded. Chondral damage to the acetabulum or femoral head was graded using the Outerbridge system [16].

The two surgeons independently and blindly assessed each other’s arthroscopic images to confirm what pathology was present and agreed in all cases. Statistical analyses were performed using the Statistical Package for the Social Sciences (SPSS) software (version 22.0, IBM SPSS, Chicago, IL, USA).

## Results

Fifty-three hip arthroscopies performed in 51 patients met the inclusion criteria from a total of 1348 hip arthroscopies performed over the 46-month period. 52 of the 53 (98%) hips included in this retrospective study had arthroscopically confirmed intra-articular hip pathology. Mean patient age was 32.5 years [15 to 67 years]) with 40 (78%) females and 11 (22%) males. There were 23 left hips and 30 right hips.

Running, Australian Rules football, gym, netball and soccer were the most common sporting activities (see Table 1 below) that patients engaged in. 21 patients did not participate in any regular sport at the time of presentation.

**Table 1:** Patient demographics

Number of patients	51 (53 hips)
Mean age (range)	32.5 (15–67)
Female: male	40:11
Left: right	23:30
Main regular sport	
Running	10
Football	3
‘Gym’‘gymnastics’	5
Netball	3
Soccer	2
Cycling	1
Hockey	1
Ice skating	1
Rowing	1
Basketball	1
Volleyball	1
Dancing	1
None/not specified	21

The most common precipitating event was pain during sport/physical activity (17/51 patients, or 33%) followed by a gradual onset of pain without an obvious precipitating event (16/51 patients or 31%)- see Table 2.

**Table 2:** Precipitating event of pain

Precipitating event	Number of cases
Sport/physical activity	17
Gradual onset (no precipitating event)	16
Unclear from clinical notes	8
Fall/slipped over	7
Childbirth	3

92.5% of the hips (49/53) were FADIR (flexion, adduction and internal rotation) positive on clinical examination, giving this test a positive predictive value of 98% (95% CI: 89.31% to 99.67%) for intra-articular pathology as all but one of the patients had arthroscopically confirmed pathology.

The most common pathology found during arthroscopy was a tear of the ligamentum teres (47/53, 89%), followed by chondral damage (35/53, 66%) and a labral tear (20/53, 38%). Damage to both the ligamentum teres and medial femoral head cartilage co-existed in 43% (23/53). Two complete tears of the LT were found during arthroscopy. Chondral damage was seen in 23 cases on the femoral head and 23 cases on the acetabulum (most commonly Outerbridge grade 1 or 2, see Table 3 below and also Additional file 2). Eleven patients had chondral damage on both the femoral head and acetabulum.

**Table 3:** Confirmed pathology

Pathology	No. of cases
No pathology	1
LT tear (partial or complete)	47
Labral tear	20
Femoral head chondral damage^a^	
Grade I	4
Grade II	15
Grade III	3
Grade IV	1
Acetabular chondral damage^a^	
Grade I	13
Grade II	8
Grade III	2
Grade IV	0

*LT* ligamentum teres

^a^I to IV, Outerbridge classification

**Fig. 1:**
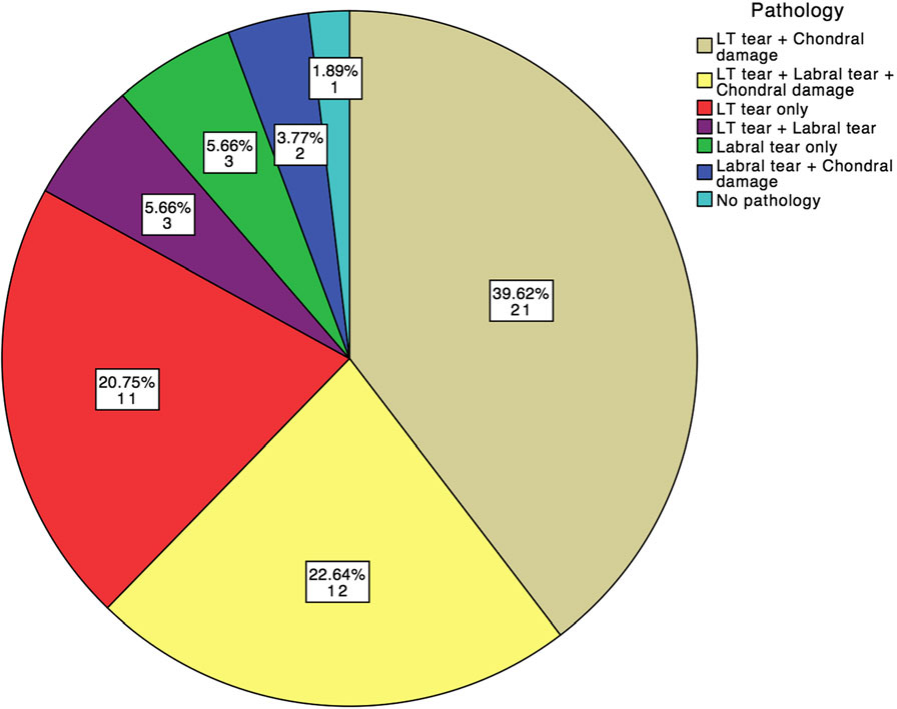
Combinations of Intra-Articular Pathology Defined at Time of Arthroscopy for Individual Patients. The most frequent pathology combination was a ligamentum teres (LT) tear with associated chondral damage, which was seen as a combination in 21 hips (40%). A combination of all 3 pathologies (LT tear, labral tear and chondral damage) co-existed in 12 hips (23%; also see Table 3 and Additional file 2)

**Fig. 2:**
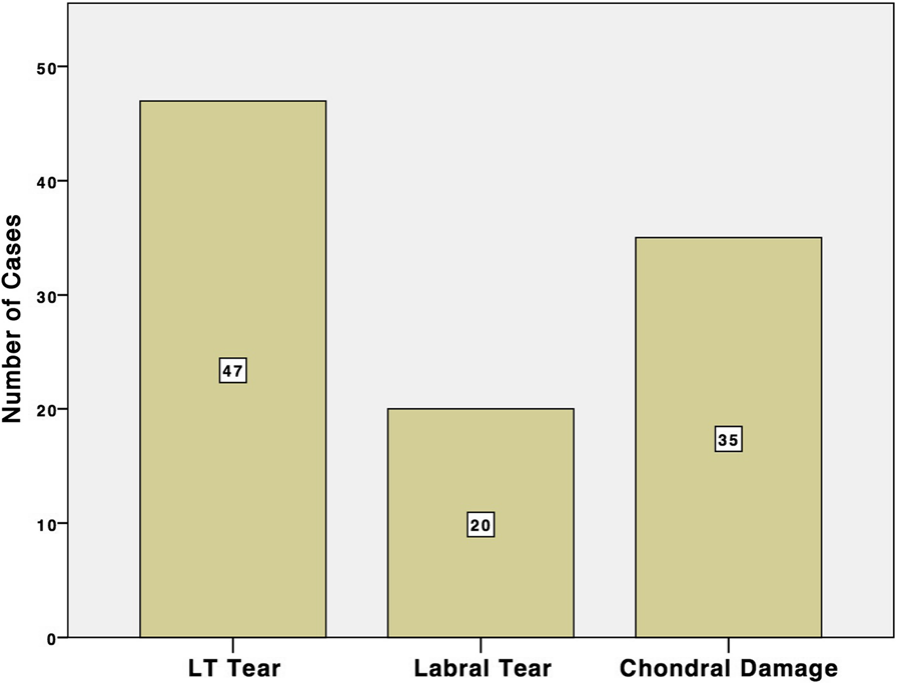
Total Proportions of Intra-Articular Pathology Identified Arthroscopically. The most common pathology arthroscopically diagnosed in patients with normal imaging was a tear of the ligamentum teres (89% of hips), followed by chondral damage (66%). Labral tears were seen in 20 hips (38%)

Labral tears were found in 20 cases (38%), and 17 of these (85%) were anterosuperior, 1 (5%) was posterosuperior and 2 (10%) extended across both anterosuperior and posterosuperior quadrants. Only 1 subject had no identifiable pathology during hip arthroscopy despite experiencing relief of pain following the intra-articular injection. Figure 1 summarizes the proportions of different combinations of pathology that were definitively diagnosed and treated arthroscopically. Figure 2 illustrates the types and incidences of intra-articular hip pathologies, with LT tears being the most common.

## Discussion

There is a very high chance of intra-articular pathology being discovered during hip arthroscopy despite normal imaging investigations in this select group of diagnostic dilemmas. With limited literature suggesting a suitable diagnostic workup for these patients, a diagnostic arthroscopy is very likely to yield pathology that *may* be the cause of a patient’s symptoms. To our knowledge, this is the first study looking at the arthroscopic findings in this select group.

The majority (89%) of the hips had a LT tear. Partial LT tears are increasingly being recognised as a source of hip pain although clinical examination and MR imaging play a limited role in detection. A LT clinical examination test has recently been described by O’Donnell et al. [17], which has been reported to have moderate to high interobserver reliability (κ coefficient 0.80) for detecting the presence of LT tears [17]. LT tears are infrequently reported or described on MRI. The incidence of reported LT tears confirmed with arthroscopy varies in the literature from 4 to 65% [14, 18–21]. The higher prevalence observed in this study may be due to the fact that only subjects with a normal MRI were included. It must also be noted that the diagnosis of LT tears is largely subjective and as previously discussed, we classified fraying and elongation of the LT as a partial tear.

A combination of pathologies were diagnosed and treated arthroscopically in 72% (38/53) of the hips. An LT tear with chondral damage of the femoral head was the most common (21/53) combination and in 9 of these cases, a labral tear was also diagnosed. This is consistent with previous literature on LT biomechanics. LT tears can lead to instability and subluxation of the hip joint with progressive damage to chondral surfaces and the labrum [22]. We observed that the majority of hips that had undergone arthroscopy for hip pain demonstrated chondropathy. Of note, chondral damage wasn’t observed as an isolated pathology.

Labral tears were found in 20 (38%) hips and most commonly co-existed with other pathology, which is consistent with previous findings [23–26] The sensitivity and specificity of MRI in the diagnosis of acetabular labral tears is 66 and 79% respectively in the literature [27]. Most labral tears were in the anterior superior quadrant, which is consistent with previous literature [28]. In this group we further postulate that LT tear/elongation and/or ligamentous instability results in repetitive anterosuperior subluxation of the femoral head in this quadrant during forced external rotation of the hip joint as a cause for this predominant soft tissue damage pattern found.

### Limitations

This study is limited by its retrospective nature, lack of control subjects and small sample size. A further limitation of this study is that MR arthrograms were not used, as standard MRIs are routine in clinical practice in this country. There is an argument for an arthrogram with dye and anaesthetic during MRI. However, we feel that a steroid injection does pose a small but significant risk of complication, which is minimised if it is used primarily for this select group after normal investigations or to decrease a large differential diagnosis. We added steroid to local anaesthetic in case there was a therapeutic effect that would limit pain to facilitate the patient’s ability to perform physical therapy prior to considering surgery. It should be again noted that the strength of the MRI magnets differed (3.0 T Vs. 1.5 T Vs. unknown) depending on where they were performed, which may have impacted on the findings.

Literature suggests that these intra-articular hip pathologies may be asymptomatic, and associated with increasing age [11, 29, 30]. However, it is important to note that all patients had failed conservative management. Collections of pre and post-operative outcome measurements were not standard practice in our clinic during the first 18 months of data collection. Patients now complete the recently validated 33-item International Hip Outcome Tool (iHOT33) [31] pre and post surgery.

## Conclusions

Although MRI is commonly used in imaging hip pathology, fluoroscopic guided injections of local anaesthetic are recommended when there is clinical suspicion, despite otherwise normal imaging. In patients with normal XR and MRI imaging but a positive response to an intra-articular injection that have failed conservative management, our study suggests that there is a 98% chance of intra-articular hip pathology being discovered on hip arthroscopy. In particular, there is a high rate of LT tears in patients with hip pain relieved by local anaesthetic and an MRI reported as normal.

## Additional files

Additional file 1: Fluoroscopic injection of steroid and local anaesthetic into the hip joint. Pain diary that patients were asked to complete. (JPG 137 kb)

Additional file 2: Observations of study parameters. Parameters including. gender, age, MRI report, response to injection and arthroscopic findings. (DOCX 145 kb)

## Abbreviations

AP: Anteroposterior
CI: Confidence interval
FADIR: Flexion, adduction and internal rotation
FAI: Femoroacetabular impingement
iHOT33 33-item: International Hip Outcome Tool
LT: Ligamentum teres
NRS: Numerical rating scale
SPSS: Statistical Package for the Social Sciences
T: Tesla
