# Supplementary figures and images for: Arthroscopic findings of a diagnostic dilemma- hip pathology with normal imaging
Source: BMC Musculoskelet Disord. 2017 Mar 21;18:120. doi: 10.1186/s12891-017-1485-5 (PMC5361835; doi:10.1186/s12891-017-1485-5)

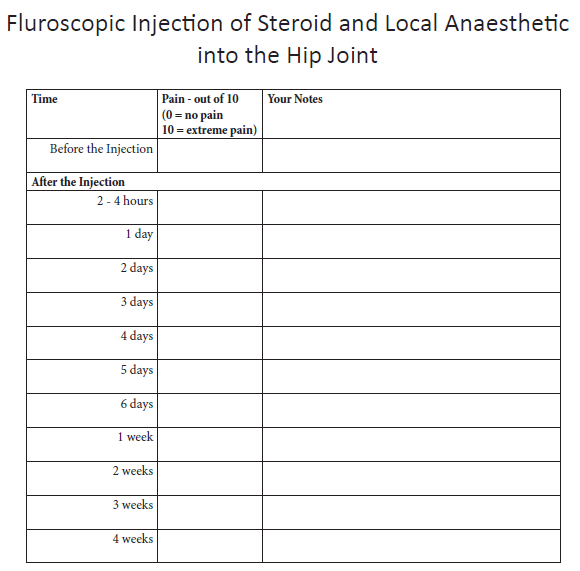

Supplement: Supplementary file 1 — Fluoroscopic injection of steroid and local anaesthetic into the hip joint. Pain diary that patients were asked to complete. (JPG 137 kb) [file 12891_2017_1485_MOESM1_ESM.jpg]
