# Supplementary material for: Arthroscopic findings of a diagnostic dilemma- hip pathology with normal imaging
Source: BMC Musculoskelet Disord. 2017 Mar 21;18:120. doi: 10.1186/s12891-017-1485-5 (PMC5361835; doi:10.1186/s12891-017-1485-5)
Supplement: Supplementary file 2 — Observations of study parameters. Parameters including. gender, age, MRI report, response to injection and arthroscopic findings. (DOCX 145 kb) [file 12891_2017_1485_MOESM2_ESM.docx]

**Additional File 2:** Observations of Study Parameters

| Case Number | Gender (age) | MRI report | Response to Injection | Arthroscopic Findings |
| --- | --- | --- | --- | --- |
| 1 | F (23) | NAD | Yes | LT 20% partial tear |
| 2 | F (38) | Mild gluteal tendinopathy | Yes | LT 15% partial tear; chondral damage (II-F)^a^ |
| 3 | F (28) | Slight pincer impingement | Yes | Labral tear; chondral damage (I-A) |
| 4 | F (28) | NAD | Yes | LT 15% partial tear |
| 5 | F (26) | NAD | Yes | LT 25% partial tear; labral tear |
| 6 | F (24) | NAD | Yes | Labral tear |
| 7 | F (39) | NAD | Yes | LT tear |
| 8 | F (28) | ?Mild labral degeneration | Yes | LT 10% partial tear; labral tear; chondral damage (I-A) |
| 9 | F (58) | NAD | Yes | LT 30% partial tear; chondral damage (III-F) |
| 10 | M (38) | NAD | Yes | LT 20% partial tear; labral tear; chondral damage (I-A) |
| 11 | F (40) | NAD | Yes | LT 20% partial tear; chondral damage (II-F) |
| 12 | F (39) | NAD | Yes | LT 30% partial tear; chondral damage (II-F) |
| 13 | F (35) | NAD | Yes | LT 20% partial tear |
| 14 | F (43) | NAD | Yes | LT 25% partial tear; chondral damage (II-F) |
| 15 | F (22) | NAD | Yes | LT partial tear |
| 16 | M (42) | Mild trochanteric bursitis | Yes | LT 30% partial tear; chondral damage (II-F) |
| 17 | F (46) | NAD | Yes | LT 20% partial tear; chondral damage (II-F) |
| 18 | F (30) | Mild trochanteric bursitis | Yes | LT 30% partial tear; chondral damage (II-F) |
| 19 | F (30) | NAD | Yes | LT 30% partial tear; chondral damage (II-F) |
| 20 | F (34) | Mild trochanteric bursitis | Yes | LT 25% partial tear; labral tear; chondral damage (I-A) |
| 21 | F (19) | NAD | Yes | LT 25% partial tear |
| 22 | F (18) | NAD | Yes | LT 30% partial tear; chondral damage (II-F) |
| 23 | M (33) | NAD | Yes | LT 75% partial tear; labral tear |
| 24 | F (52) | NAD | Yes | LT partial tear; chondral damage (II-F) |
| 25 | M (31) | Mild adductor tendinopathy | Yes | LT partial tear; chondral damage (I-F) (I-A) |
| 26 | F (46) | NAD | Yes | LT partial tear |
| 27 | M (67) | NAD | Yes | LT partial tear; chondral damage (II-F) (II-A) |
| 28 | F (17) | NAD | Yes | LT partial tear |
| 29 | F (17) | Small hip effusion, otherwise NAD | Yes | LT 75% partial tear; chondral damage (II-F) (I-A) |
| 30 | F (41) | Absent LT, small effusion | Yes | LT complete tear; labral tear; chondral damage (III-F) (I-A) |
| 31 | F (39) | NAD | Yes | LT partial tear; labral tear; chondral damage (I-A) |
| 32 | M (15) | NAD | Yes | LT 20% partial tear |
| 33 | F (16) | NAD | Yes | LT 95% partial tear; chondral damage (I-F) |
| 34 | F (38) | Gluteus medius tendinopathy | Yes | LT 75% partial tear; labral tear; chondral damage (IV-F) (I-A) |
| 35 | F (62) | NAD | Yes | LT partial tear; chondral damage (I-A) |
| 36 | F (39) | NAD (?occult labral tear) | Yes | LT 30% partial tear; chondral damage (II-F) (II-A) |
| 37 | F (35) | NAD | Yes | LT 75% partial tear, chondral damage (I-F) (II-A) |
| 38 | M (35) | Gluteus medius tendinosis | Yes | LT partial tear, labral tear, chondral damage (II-F) (II-A) |
| 39 | F (43) | Gluteus medius and minimus tendinosis | Yes | LT elongation, labral tear |
| 40 | F (23) | NAD | Yes | Labral tear |
| 41 | M (41) | NAD | Yes | Labral tear, chondral damage (II-A) |
| 42 | M (24) | NAD | Yes | LT 10% partial tear, labral tear, chondral damage (I-A) |
| 43 | F (22) | NAD | Yes | LT 10% partial tear |
| 44 | M (35) | NAD | Yes | LT 60% partial tear, labral tear, chondral damage (II-A) |
| 45 | F (26) | NAD (small effusion) | Yes | LT 20% partial tear, labral tear, chondral damage (II-A) |
| 46 | M (27) | NAD | Yes | LT 30% partial tear, chondral damage (I-A) |
| 47 | F (31) | NAD (very mild gluteus minimus peritendon synovitis) | Yes | No pathology found |
| 48 | F (32) | NAD | Yes | LT 20% partial tear |
| 49 | F (34) | Mild bursitis, otherwise NAD | Yes | Labral tear |
| 50 | F (28) | NAD | Yes | LT 10% partial tear, labral tear, chondral damage (II-F) (I-A) |
| 51 | F (31) | NAD | Yes | LT 75% partial tear, chondral damage (III-F) (III-A) |
| 52 | F (20) | NAD | Yes | LT complete tear, chondral damage (I-F) (III-A) |
| 53 | F (25) | NAD (muscle contusion) | Yes | LT partial tear, labral tear, chondral damage (II-A) |
